# Increases in negative affective arousal precede lower self-esteem in patients with borderline personality disorder but not in patients with depressive disorders: an experience sampling approach

**DOI:** 10.1186/s40479-023-00229-w

**Published:** 2023-10-03

**Authors:** Johannes Bodo Heekerens, Lars Schulze, Juliane Enge, Babette Renneberg, Stefan Roepke

**Affiliations:** 1 Department of Psychiatry and Neurosciences, Charité - Universitätsmedizin Berlin, Berlin, Germany; 2 Department of Education and Psychology, Clinical Psychology and Psychotherapy, Freie Universität Berlin, Habelschwerdter Allee 45, Berlin, 14195 Germany

**Keywords:** Borderline personality disorder, Depression, Self-esteem, Affect, Experience sampling, Dynamic structural equation modeling

## Abstract

**Background:**

Instability in self-esteem and instability in affect are core features of borderline personality disorder (BPD). For decades, researchers and theorists have been interested in the temporal dynamics between these constructs. Some hypothesize that changes in affective states should precede changes in self-esteem (Linehan, Cognitive-behavioral treatment of borderline personality disorder. Diagnosis and treatment of mental disorders, 1993), while others suggest that changes in self-esteem should precede changes in affective states (Kernberg, Borderline conditions and pathological narcissism, 1975).

**Methods:**

In this study, we investigated the temporal relations between negative affective arousal states and current self-esteem in daily life. Patients with BPD (*n* = 42) or depressive disorders (DD; *n* = 40), and non-clinical controls (NCC; *n* = 40) were assessed every 15 min for 13 h.

**Results:**

As expected, dynamic structural equation modeling showed higher levels of average daily negative affective arousal and lower levels of average daily self-esteem in the BPD group compared with the NCC group, and scores in the DD group were in-between the BPD and the NCC groups. In line with predictions based on Linehan’s (Cognitive-behavioral treatment of borderline personality disorder. Diagnosis and treatment of mental disorders, 1993) model of affective dysregulation in BPD, negative affective arousal (t) and subsequent self-esteem (t+ 1) were significantly linked only in the BPD group, implying that higher negative affective arousal is followed by lower current self-esteem in the next measurement (ca. 15 min later). Importantly, self-esteem (t) and subsequent negative affective arousal (t + 1) were not significantly related (Kernberg, Borderline conditions and pathological narcissism, 1975).

**Conclusions:**

Our findings suggest close dynamic temporal relations between affective instability and self-esteem instability in BPD, which highlights the importance of providing patients with means to effectively modulate high negative affective arousal states.

**Supplementary Information:**

The online version contains supplementary material available at 10.1186/s40479-023-00229-w.

## Background

Instability in self-esteem and instability in affect are core features of borderline personality disorder (BPD) [3, 84]. Self-esteem is an aspect of identity and is defined as overall feelings towards oneself as worthy or unworthy [65]. Numerous studies have investigated self-esteem in patients with BPD (see [13] for a recent review). A large body of evidence suggests that patients with BPD report significantly lower levels of self-esteem compared with non-clinical controls, as well as heightened levels of instability in self-esteem [42, 68–70]. In line with this, the alternative DSM-5 model for personality disorders lists an unstable self (including instability of self-esteem) as an essential feature of impairments in personality functioning [3]. In addition, a wealth of studies found that patients with BPD report significantly higher levels of and instability in negative affective arousal than non-clinical controls ([15, 16]; see [66] for a review). Researchers hypothesized that instability in self-esteem closely relates to instability in negative affective arousal states, but various theoretical perspectives have suggested different temporal links between the constructs: Linehan [44] argued that instability in self-esteem is an affective (dys-)regulation problem, while Kernberg [38] explained that instability in self-esteem leads to negative affective states (including negative states of high affective arousal) that are difficult to regulate. In this study, we investigate the temporal links between negative affective arousal and self-esteem in BPD.

### Temporal links between affect and self-esteem in BPD

In line with Linehan’s [44] premise that instability in self-esteem is an affective (dys-) regulation problem, past research has demonstrated that low levels of self-esteem relate to physiological stress parameters and that this association may be exacerbated by individuals’ inability to manage and regulate (negative) affective states (see [14, 24] for reviews). In addition, initial diary studies in student samples found higher levels of negative affect in response to everyday stressors on days with lower levels of self-esteem [26, 39], and some studies suggest that this association is stronger in individuals with heightened levels of BPD features ([78, 85], also see [63]). Other studies in non-clinical population linked low levels of self-esteem to affective instability [18, 40], as well as choice of less effective strategies to regulate emotions [32, 74]. One e-diary study found that momentary (negative) affective arousal states and low levels of daily average self-esteem separately predicted nonsuicidal self-injuries in patients with BPD [67, 68]. However, very few studies directly tested Linehan’s [44] theoretical premise by investigating the dynamic temporal relations between changes in current self-esteem and affective arousal states. If identity disturbances in BPD indeed arise from difficulties in emotion regulation, drops in current self-esteem should primarily occur after changes in affective states that individuals fail to adequately modulate. Consistent with this idea, patients with BPD are thought to identify fully with momentary affective states, which may explain why affective states strongly influence self-evaluations ([23]; also see [44]). In other words, how patients with BPD feel towards themselves largely depends on current affective experiences and if these affective experiences happen to be negative, as they frequently are, unfavorable self-evaluations and further increases in negative affective arousal follow. Patients with BPD have been shown to excessively ruminate on negative affective states, which gives rise to negative self-related thoughts and increased levels of negative affective arousal [73]. Related to this, it has been argued that a defining feature of BPD is the inability to form mental images that help to perceive and interpret own and others intentional mental states (e.g., affects), which gives rise to the assumption that affective states are direct representations of psychical reality such as oneself and this may explain why patients with BPD rely on current affective states as a basis for self-evaluation [21].

On the other hand, Kernberg [38] has argued that instability in self-esteem, which reflects a disturbed identity, leads to negative affective states that are difficult to regulate (also see [72]). Specifically, exploring various identity elements to commit to a set of personally meaningful goals, values, and beliefs (identity formation) is thought to be taxing and has been associated with negative affect [47, 48]. Patients with BPD experience sharp discontinuities in goals, values, and beliefs, which may make them prone to uncertainties associated with lack of commitments to important life issues that, in turn, lead to negative self-evaluations that have also been argued to give rise to existential anxiety [58] and promote feelings of helplessness [38]. In line with this, studies found that patients with BPD report lower levels of self-concept clarity than non-clinical controls [57, 64]. There is also evidence that identity incoherence (feelings of distress about lacking a coherent sense of self) and lack of commitment (to goals or a constant set of values) closely relate to affective dysregulation in BPD [83]. Two e-diary studies in patients with BPD investigated the interplay of instability in negative affective arousal and instability in self-esteem using multiple momentary self-report ratings in everyday life. Results of the first study show that indices of affective instability and self-esteem instability were positively correlated in patients with BPD but not in non-clinical controls ([70]; also see [66, 69]). The authors also found a positive relation between squared successive differences in self-esteem reports and later squared successive differences in arousal and valence dimensions of affect, suggesting that changes in self-esteem predict subsequent changes in negative affective arousal states. At first glance, this is in line with Kernberg’s [38] prediction. However, the method does not allow to determine whether increases or decreases in self-esteem relate to affect, and the authors do not report results on a plausible reverse effect linking changes in affect to subsequent changes in self-esteem (more in line with [44]). The second study was a follow-up and included a clinical comparison group, demonstrating that while instability in affect was comparable across patients with BPD and patients with anxiety disorders, instability in self-esteem was particularly prominent in patients with BPD [42].

To date it remains largely unclear how changes in (negative) affective arousal relate to changes in self-esteem as, to the best of our knowledge, only one study directly examined the temporal relation between affective instability and self-esteem instability [70]. The study used hourly assessments and showed that decreases in self-esteem translate into (negative) affective arousal over the course of hours. It was not designed to detect swift dynamic processes, which requires dense sampling plans [15]. Thus, we used 15 min assessment intervals in this study and investigate to which degree (a) changes in (negative) affective arousal influence self-esteem over time and (b) changes in self-esteem influence (negative) affective arousal over time. Findings from this study may help to further develop clinical interventions [64]. For example, if evidence suggests that increases in affective arousal precede reductions in self-esteem, therapists should encourage patients with BPD to implement functional affect regulation strategies, or, if decreases in self-esteem are found to precede increases in affective arousal, patients may be instructed to focus their attention on bolstering current self-esteem.

### Study aim and hypotheses

In the present study we examined the temporal relations between (negative) affective arousal and current self-esteem in patients with BPD and compared these with those in patients with depressive disorders (DD) and non-clinical controls (NCC). The clinical comparison is crucial to investigate whether potential temporal relations are (phenomena) specific to BPD. Patients with depressive disorders and patients with BPD both report low levels of self-esteem, high levels of negative affective arousal, and elevated levels of affective instability compared with non-clinical controls (e.g., Trull et al. 2008 [79], [50]; see [13, 81], Sowislo and Orth 2013 for reviews [76]). Lower levels of self-esteem and self-esteem instability were also found to relate to more depressive symptoms (e.g., [39, 56, 77]). Thus, we hypothesized that (1 patients with BPD report higher daily average levels of negative affective arousal and lower daily average levels of self-esteem than patients with DD, which in turn report higher daily average levels of negative affective arousal and lower daily average levels of self-esteem than NCC would,(2 increases in negative affective arousal would predict decreases in self-esteem at the subsequent measurement occasion in patients with BPD, and to a lesser degree in patients with DD, but not in NCC (based on [44]), (3) decreases in self-esteem would predict increases in negative affective arousal at the subsequent measurement occasion in patients with BPD, and to a lesser degree in patients with DD, NCC (based on [38]).

## Method

### Data transparency and availability

Data for this study were collected as part of a larger data set. Participants provided data during three individual assessments and the rest of these data were reported in separate manuscripts that focused on temporal relations between negative affective arousal and perceived rejection (first assessment; [29]), as well as between arousal and valence dimensions of affect and dissociation (second assessment; [30]). In the current manuscript, we focus on temporal relations between negative affective arousal and self-esteem (third assessment). Participants overlapped between those studies. However, individuals included in this study are not identical with our past studies [29, 30].

The published manuscripts [29, 30] include detailed descriptions of the data collection process and study procedures. We only included the most important and new information in the current manuscript. The anonymized data set, statistical code, and additional results are available at: https://osf.io/8xwtc/.

### Sample

The initial sample comprised 49 patients with BPD, 47 patients with DD, and 49 NCCs. Participants were at least 18 years old and were interviewed using the German version of the Structured Clinical Interview for DSM-IV Axis I Disorders (SCID-I) and Axis II Disorders (SCID-II; [20, 82]; see [29, 30] for details). In the DD group, participants had to meet DSM-IV diagnostic criteria for a current or chronic depressive disorder (see Table S1 in the online supplement materials for further details). NCC participants were only included if they were not taking psychotropic medication and had no current nor lifetime diagnosis of mental or neurological disorders (e.g., traumatic diseases of the central nervous system). Exclusion criteria for participants with BPD and DD were comorbid diagnosis of past or present psychotic disorder, bipolar disorder, cognitive disorders (e.g., delirium, dementia), neurological disorders, and substance-associated disorders. After data collection, we excluded participants who answered less than 10 out of 52 during the e-diary phase to ensure a minimum number of valid responses prompts (*n* = 6 in BPD group, *n* = 7 in DD group, and *n* = 8 in NCC group). In addition, respectively one participant in the BPD and NCC group was excluded due to zero variance in the e-diary items.^1^ The final sample comprised 42 patients with BPD, 40 patients with DD (21 outpatients and 19 inpatients), and 40 NCCs.

BPD and DD inpatients were recruited at the Department of Psychiatry and Neuroscience, Charité – Universitätsmedizin Berlin. All patients with BPD underwent a planned 12-week inpatient dialectical behavioral treatment program or a 2-week preparing diagnostic stay in the Department of Psychiatry and Neuroscience (Charité – Universitätsmedizin Berlin, Germany). All other participants were recruited via public advertising. The Fisher`s exact test indicated no significant differences between groups regarding gender; 35/42 participants were female in the BPD group compared with 27/40 participants in the DD group and 31/39 participants in the NCC group (*p* = 0.247). No participant indicated a non-binary gender identity (e.g., bigender, genderfluid). There was no significant difference in age between the groups; the median (range) age was 31 (18–54) in the BPD group, 34 (18–60) in the DD group, and 30 (18–50) in the NCC group (*F*(2, 119) = 2.03, *p* = 0.136). The chi-square test indicated no differences in the proportion of participants receiving psychotropic medication between the BPD group (27/42, 64.29%) and the DD group (27/40, 67.50%) (${\chi }^{2}$(1, *N* = 82) = 0.01; *p* = 0.941; OR = 1.15; 95% CI [0.46, 2.88]). The most frequent comorbid diagnoses were PTSD (14/42 in BPD compared to 4/40 in DD) and eating disorders (11/42 in BPD compared to 3/40 in DD) (see Table S1 in the online supplement materials for all comorbid diagnoses). The chi-square test revealed a significant difference in the number of participants with PTSD between groups (${\chi }^{2}$(1, *N* = 82) = 5.22; *p* = 0.022; OR = 4.50; 95% CI [1.33, 15.18]). All participants were informed about the voluntary nature of their participation and data protection. Informed consent was signed prior to the start of the study (see document S2 in the online supplement materials for the original file and a translation). The institutional review board (Charité - Universitätsmedizin Berlin, Germany) approved the study (No. EA4/138/15).

### Measures

Prior to the e-diary assessment participants completed diagnostic instruments in rooms of the university clinic. We calculated mean scores for all questionnaires (also see Table 1).

**Table 1:** Mean, standard deviations, and 95% confidence intervals of mean differences for the BSL-95, QTF, BDI-II, and SCL90R in the BPD (*n* = 42), DD (*n* = 40) and NCC (*n* = 40) groups

Measure	M	SD	BPD vs DD: [95% CI] Diff	*p*	BPD vs HC: [95% CI] Diff	*p*	DD vs HC: [95% CI] Diff	*p*
Borderline Personality Disorder (BPD)								
1. BSL-95	1.97	0.55	0.11 [-0.24, 0.46]	.735	**0.52 [0.17, 0.86]**	**.002**	**0.41 [0.06, 0.75]**	**.017**
2. QTF	2.66	0.56	**0.85 [0.43, 1.26]**	**.001**	**1.37 [0.96, 1.79]**	**.000**	**0.53 [0.12, 0.93]**	**.000**
3. BDI-II	2.70	1.23	**1.46 [1.00, 1.92]**	**.000**	**2.42 [1.96, 2.88]**	**.000**	**0.95 [0.58, 1.33]**	**.011**
4. GSI	2.03	0.55	0.15 [-0.20, 0.49]	.583	**0.75 [0.41, 1.10]**	**.000**	**0.61 [0.26, 0.96]**	**.000**
Depressive Disorders (DD)								
1. BSL-95	1.86	0.88						
2. QTF	1.81	1.09						
3. BDI-II	1.23	0.56						
4. GSI	1.89	0.90						
Non-Clinical Control (NCC)								
1. BSL-95	1.46	0.43						
2. QTF	1.28	0.49						
3. BDI-II	0.28	0.42						
4. GSI	1.28	0.39						

Significant differences are in bold

*M* mean, *SD *standard deviation, *p p*-value obtained using Tukey’s HSD tests, *Diff *mean difference, *CI* confidence interval obtained using Tukey’s HSD tests, *BSL-95 *Borderline Symptoms List, *QTF *Thoughts and Feelings Questionnaire, *BDI-II *Beck Depression Inventory, *GSI SCL-90R* Global Severity Index

#### Questionnaires

We assessed the intensity of BPD specific cognitions using the German Questionnaire for Thoughts and Feelings (QTF; 14 statements; e.g., “close interpersonal relationships are threatening”; range 1–5; [61, 62]). McDonald’s [49] hierarchical omega was 0.92, 95% CI [0.88, 0.95] (computed using maximum likelihood robust as implemented in the R package MBESS; [36]).^2^ In addition, severity of depressive symptoms was assessed using the German version of Beck’s Depression Inventory (BDI-II; 21 items; e.g., sadness in the past 2 weeks; range 0–3; [6, 31]). Hierarchical omega was 0.95, 95% CI [0.93, 0.97]. The 95-item version of the German Borderline Symptom List (BSL-95; [7]) was used to assess baseline severity of BPD psychopathology (e.g., “during the past week I thought about hurting myself”, range 0–4). Hierarchical omega for the self-regulation subscale was 0.94, 95% CI [0.92, 0.96], affect regulation subscale 0.92, 95% CI [0.89, 0.94], self-destruction subscale 0.91, 95% CI [0.84, 0.94], dysphoria subscale 0.92, 95% CI [0.88, 0.94], hostility subscale 0.71, 95% CI [0.58, 0.80], and intrusions subscale 0.85, 95% CI [0.78, 0.92]. Finally, we used the revised German 90-items version of the Symptom Checklist (SCL-90R; [11, 22]) to assess the severity of several psychopathological symptoms (e.g., nervousness in the past week, range 0–4). Hierarchical omega for the somatization subscale was 0.88 95% CI [0.82, 0.91], obsessive compulsion 0.85, 95% CI [0.79, 0.89], interpersonal sensitivity 0.88, 95% CI [0.83, 0.91], depression 0.92, 95% CI [0.90, 0.94], anxiety 0.80, 95% CI [0.68, 0.87], aggression 0.73, 95% CI [0.61, 0.81], phobic anxiety 0.83, 95% CI [0.77, 0.88], paranoid ideation 0.79, 95% CI [0.70, 0.85], and psychoticism 0.83, 95% CI [0.76, 0.91].

#### E-diary assessment

E-diary data were collected during patients’ daily clinic routine or daily lives between July 2016 and September 2018 (see [29, 30] for details). The e-diary emitted a prompting signal according to a time-sampling schedule in intervals of 15 min (± 5 min) from 8 am to 9 pm (e.g., [15, 41]). Participants were prompted 52 times within 1 day. Each response was automatically time-stamped. At each prompt participants rated their current affective arousal and current self-esteem. Specifically, participants rated current levels of “Anspannung” (German for “tension”) using a visual analog scale ranging from 0 (“*no arousal at all*”) to 10 (“*extreme arousal*”). The word “Anspannung” in connection with affective states is negatively connoted and indicates negative affective arousal. Current self-esteem was assessed using items borrowed from the Rosenberg Self-Esteem Scale [65]. Previous experience sampling studies used a four-items scale based on the Rosenberg Self-Esteem Scale (e.g., Nezlek and Kuppens 2008 [55], [42, 70]). In this study, we used a two-items scale to reduce the burden placed on participants. The English (translated) wording was: “At the moment, I think I am a valuable person” and “At the moment, I think I am worthless” (reverse coded). The German (original) wording was: “Im Moment denke ich, ich bin ein wertvoller Mensch” and “Im Moment denke ich, ich bin wertlos” (reverse coded). The items conceptually relate to item 7 (“I feel that I’m a person of worth”) in the Rosenberg Self-Esteem Scale. The items are similar to items used in Santangelo et al. [70] (e.g., “At the moment, I am satisfied with myself” and “At the moment, I think I am no good at all”). In our study, participants rated the items using a visual analog scale ranging from 0 (“*not at all*”) to 10 (“*completely*”). We built a composite score reflecting the mean value for each measurement occasion across both items and used this score for all further analyses (as done in previous studies; e.g., [70]). Multilevel composite reliability ([43], also see [25]) for the overall score was 0.89, 95% CI [0.86, 0.92] (Bayesian credible intervals computed using M*plus*; see [51] for technical details). Reliability of the composite score was 0.58, 95% CI [0.53, 0.62] on the within-level and 0.92, 95% CI [0.88, 0.94]^3^ on the between- level. Because the within-level estimate indicates moderate reliability, we ran additional models using single-item indicators for feeling of worth and feeling worthless to investigate potential differences (see Sensitivity and additional analyses section).

### Statistical analysis

**Fig. 1:**
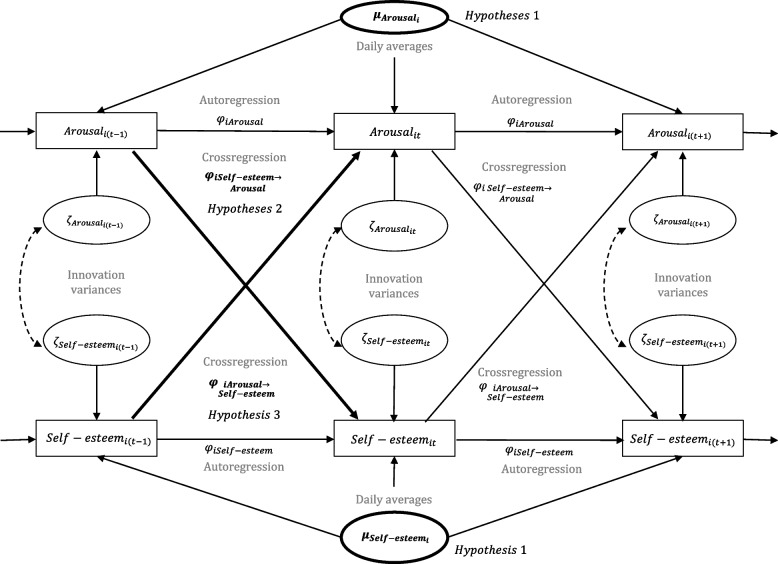
Dynamic structural equation modeling of arousal and self-esteem. Symbols in bold indicate effects relevant for our tests of hypotheses. Arousal and self-esteem are decomposed into their respective between-person (${\mu }_{A{rousal}_{i}}$ and ${\mu }_{{Self-esteem}_{i}}$) and within-person (${\mu }_{A{rousal}_{it}}$ and ${\mu }_{{Self-esteem}_{it}}$) parts. The between-person parts reflect the daily average scores for arousal and self-esteem across all time points *t* for participant *i*. On the within-level, the model includes random autoregressive effects (*φ*_*iArousal*_ and *φ*_*iSelf*−*esteem*_) and random cross-lagged effects (ϕiAonS and ϕiSonA) of order 1 on the within-person level. The autoregressive effects reflect the average daily regression coefficient of arousal and arousal at the next time point (*φ*_*iArousal*_) or self-esteem and self-esteem at the next time point (*φ*_*iSelf*−*esteem*_) for participant *i*. The cross-lagged effects reflect the average daily regression coefficient of self-esteem and arousal at the next time point (*φ*_*iSelf*−*esteem*→*Arousal*_) or arousal and self-esteem at the next time point (*φ*_*iArousal*→*Self*−*esteem*_) for participant *i*. In addition, we modeled the variance of the innovation terms of arousal (${\zeta }_{A{rousal}_{it}}$) and self-esteem (${\zeta }_{{Self-esteem}_{it}}$) as random effects. Innovation variances depict unexplained variance due to measurement error and interindividual differences in the reactivity to unobserved influences

We analyzed our data using dynamic structural equation models (dynamic SEM; [4]) that separate interindividual between-person differences (“traits”) from intraindividual within-person fluctuations (“states”) around this value. Dynamic SEMs allow to investigate interindividual differences in intraindividual processes such as autoregressive and cross-lagged associations. All models presented here estimate the latent person-specific means of arousal and self-esteem across measurement occasions (daily average levels or “traits”). These estimates are then averaged across all participants, as well as across participants in the diagnostic groups (BPD, DD, or NCC) in separate models. In addition, the models include autocorrelations of order 1 (AR[1]) and cross-lagged effects of current arousal and current self-esteem at the within-person level (see [27] for a similar model). The autoregressive associations of order 1 describe the degree to which arousal (or self-esteem) states are predictive of arousal (or self-esteem) states on the following measurement occasion (ca. 15 min later). The cross-lagged associations describe the degree to which self-esteem (or arousal) states are predictive of arousal (or self-esteem) states on the following measurement occasion (ca. 15 min later). Autoregressive and cross-lagged effects were averaged across all participants, as well as across participants in the diagnostic groups (BPD, DD, or NCC) in separate models (fixed effects). The models also estimate person-specific deviations from these averages (random-effects variances). The models also allowed for person-specific random innovation variances (i.e., level 1 residual variances) that capture interindividual differences in the exposure and reactivity to unobserved influences (see [33] for a thorough discussion). A graphical display of the model used in our analyses is shown in Fig. 1.

To test our hypotheses, we calculated four separate models. In our first model, a grouping variable (BPD = 1, DD = 2, NCC = 3) was used to predict differences in daily average (“trait”) levels of arousal and self-esteem, as well as to predict differences in autoregressive and cross-lagged parameters. Group-specific model parameters were computed using three additional models based on responses from participants in the BPD, DD, or NCC group only (see Heekerens et al. [29] for a similar approach). The data set used for our analyses includes 52 rows for each participant, which corresponds to the number of quarter-hourly prompts in the time between 8 am and 9 pm (as recommended by [4]). For omitted prompts, missing observations (i.e., empty rows) were inserted in the data set to ensure equidistant measurements. For example, if a person answered a prompt at 9.15 AM and the next prompt at 10.00 AM, we would set the prompts at 9.30 AM and 9.45 AM to missing. Missing observations are filled with values generated from available information and a multiple imputation model in the context of this algorithm (see Aspharouhov and Muthén 2010 for technical details [5]). Dynamic SEM uses Bayesian methods and the Markov Chain Monte Carlo algorithm based on the Gibbs sampler [4]. All Bayesian analyses were conducted using the M*plus* default priors. Because the success of the Gibbs sampler estimation process depends on correctly diagnosing convergence to construct the posterior distributions of parameters, we carefully checked model convergence. We assumed convergence if the potential scale reduction factor fell below the M*plus* default cut-off of 1.10 for all parameters. We used M*plus* 8.7 [52] to compute the multilevel reliability coefficients, estimate the models for our hypothesis tests, and for sensitivity and additional analyses. Preliminary analyses and all other reliability estimates were computed using R version 4.2.1 [59].

Prior to testing our hypotheses, we conducted ANOVAs to see whether baseline questionnaire scores differed between the three groups. As expected, there were significant group differences in severity of borderline symptoms (*F*(2,115) = 7.02; *p* = 0.001; ω = 0.11; 95% CI [0.03, 0.19]; BPD = DD > NCC), depressive symptoms (*F*(2,96) = 79.10; *p* < 0.001; ω = 0.61; 95% CI [0.52, 0.69]; BPD > DD > NCC), borderline-specific cognitions (*F*(2,113) = 31.84; *p* < 0.001; ω = 0.35; 95% CI [0.24, 0.45]; BPD > DD > NCC), and general psychological symptoms (*F*(2,115) = 15.15; *p* < 0.001; ω = 0.21; 95% CI [0.10, 0.30]; BPD = DD > NCC). Results of the Tukey’s HSD tests of differences between the diagnostic groups (BPD, DD, NCC) are presented in Table 1.

## Results

### Preliminary analyses

Because we allowed participants to enter responses on demand, we were unable to calculate a compliance rate based on the proportion of answered prompts. Instead, we calculated the proportion of valid responses within the 13-h assessment period. A valid response was defined as the first entry within a 15 min period (± 5 min) starting at 8 am and ending at 9 pm. The maximum number of valid responses was 52. The average realized number of responses was 23.50 (45.19%, *SD* = 8.26) in the BPD group, 28.05 (53.94%, *SD* = 10.21) in the DD group, and 28.45 (54.71%, *SD* = 8.89) in the NCC group. Across groups, most missing responses occurred in the morning between 9 and 10 AM (see document S3 in the online supplement materials for details). According to scale reduction factor values and visual inspection of trace plots, all dynamic SEMs converged well.

### Test of hypotheses

#### Daily average arousal and self-esteem ratings across diagnostic groups

According to our first hypothesis, we expected patients with BPD to report the highest trait levels of arousal and the lowest levels of self-esteem. Arousal and self-esteem were rated on scales from 0 to 10. As predicted, descriptive results (see Table 2) show that patients in the BPD group reported the highest average (“trait”) arousal ratings. Arousal in the BPD group was5.86, 95% CI [5.57, 6.14], compared to 3.42, 95% CI [2.70, 4.16] in the DD group, and 1.91, 95% CI [1.43, 2.41] in the NCC group. Patients with BPD also report the lowest average (“trait”) self-esteem ratings. Self-esteem in the BPD group was 3.22, 95% CI [2.45, 3.93], compared to 5.18, 95% CI [4.16, 6.17] in the DD group, and 8.93, 95% CI [8.55, 9.27] in the NCC group. Accordingly, group was a significant predictor of differences in trait arousal (-1.98, 95% CI [-2.30, -1.65]) and trait self-esteem (2.77, 95% CI [2.77, 3.26]).^4^ Further analyses confirmed that scores in the BPD group significantly differed from scores in the DD group, which in turn significantly differed from scores in the NCC group (arousal: BPD > DD > NCC; self-esteem: BPD < DD < NCC) (see Sensitivity and additional analyses section for details).

**Table 2:**
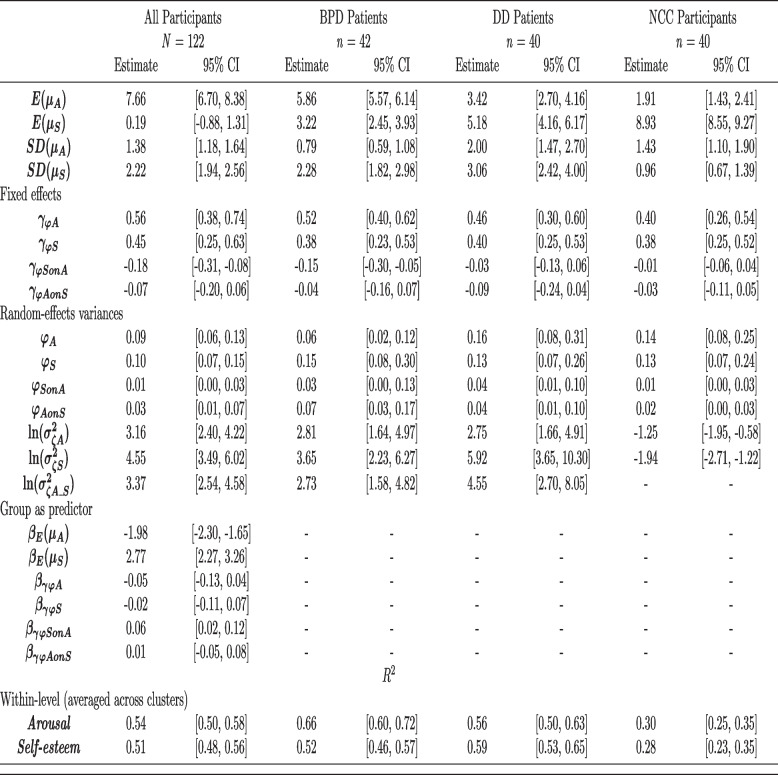
Results of the dynamic SEMs of the relations between arousal (A) and self-esteem (S) in patients with borderline personality disorder (BPD), depressive disorders (DD) and non-clinical controls (NCC)

*SEM* Structural equation model. All parameters are unstandardized parameters and denote posterior medians. The 95% CIs denote Bayesian credibility intervals. *R*^2^ measures refer to explained variance in arousal and self-esteem on the within-level. Arousal and self-esteem were assessed on 0 to 10 scales. Group (1 = BPD, 2 = DD, 3 = NCC). *µ*_*A*_ = person-specific mean of arousal across measurement occasions, *µ*_*S*_ = person-specific mean of self-esteem across measurement occasions, *γ*_*φA*_ = average autoregressive effect (fixed effect) of arousal, *γ*_*φD*_ = average autoregressive effect (fixed effect) of self-esteem, *γ*_*φSonA*_ = average slope (fixed effect) of the regressions of self-esteem on arousal at time *t*-1, *γ*_*φAonS*_ = average slope (fixed effect) of the regressions of arousal on self-esteem at time *t*-1, *φ*_*A*_ = random, person-specific autoregressive effect of arousal, *φ*_*S*_ = random, person-specific autoregressive effect of self-esteem, *φ*_*SonA*_ = random, person-specific slope of the regressions of self-esteem on arousal at time *t*-1, $\mathrm{ln}({\sigma }_{\zeta A}^{2})$ = logarithm of the random, person-specific innovation variance of arousal (including measurement error), $\mathrm{ln}({\sigma }_{\zeta S}^{2})$ = logarithm of the random, person-specific innovation variance of self-esteem (including measurement error). $\mathrm{ln}({\sigma }_{\zeta A\_S}^{2})$ = mean of the log of covariance between the random innovations of arousal and self-esteem at the same time point

#### Cross-lagged temporal relations across diagnostic groups

According to our second hypothesis, we expected a significant cross-lagged relation between higher arousal (t) and lower self-esteem at the following measurement (t + 1) in patients with BPD and patients with DD. As shown in Table 2, the unstandardized (fixed) effect that regresses arousal (t) on self-esteem at the following measurement (t + 1; ca. 15 min later) was estimated at -0.15, 95% CI [-0.30, -0.05], in the BPD group. In other words, we found a negative relation between current levels of arousal and later self-esteem, implying that momentary arousal above a person’s daily average precede lower self-esteem ratings. As predicted, the effect was significant in the BPD group. In contrast to our hypotheses, the effect was not significant in the DD group, -0.03, 95% CI [-0.13, 0.06]. In the NCC group the effect was -0.01, 95% CI [-0.06, 0.04]. In a model including responses from all participants, group was a significant predictor of differences in the cross-lagged relation between arousal (t) and self-esteem at the following measurement (t + 1), 0.06, 95% CI [0.02, 0.12]. Further analyses confirmed that the cross-lagged association of current arousal on later self-esteem was significantly higher in the BPD group significantly compared to the DD and NCC group. There was no significant difference between the DD and NCC groups (BPD > DD = NCC) (see Sensitivity and additional analyses section for details).

According to our third hypothesis, we expected a significant cross-lagged relation between lower self-esteem and higher arousal at the following measurement in patients with BPD and patients with DD. As shown in Table 2, the unstandardized (fixed) effect that regresses arousal on self-esteem on the previous assessment (15 min earlier) was estimated at -0.04, 95% CI [-0.16, 0.07], in the BPD group and -0.09, 95% CI [-0.24, 0.04] in the DD group. Other than expected, the (fixed) effects did not reach statistical significance. In the NCC group, the effect was -0.03, 95% CI [-0.11, 0.05]. In a model including responses from all participants, group was not a significant predictor of differences in the cross-lagged relation between self-esteem and lag1 arousal, 0.01, 95% CI [-0.05, 0.08] (BPD = DD = NCC).

#### Autoregressive temporal relations across diagnostic groups

Measurements in this study were ca. 15 min apart and we expected substantial autoregressive relations, although we did not formulate hypotheses regarding autoregressive effects. As shown in Table 2, the unstandardized (fixed) effect that regresses arousal on the previous measurement was 0.52, 95% CI [0.40, 0.62], in the BPD group. The fixed effect for self-esteem was estimated at 0.38, 95% CI [0.23, 0.53], in the BPD group. Thus, we found the expected positive relations between current and later measurements of arousal and self-esteem. Table 2 shows that similar effects were found in the DD and NCC groups. In a model including responses from all participants, group was neither a significant predictor of differences in the autoregressive effect for arousal, -0.05, 95% CI [-0.13, 0.04], nor in the autoregressive effect for self-esteem, -0.02, 95% CI [-0.11, 0.07] (BPD = DD = NCC).

### Sensitivity and additional analyses

To test the robustness of our effects, we conducted several sensitivity and additional analyses. First, we computed three dynamic SEMs including dummy-coded variables as predictors to separately investigate differences when directly comparing the BPD and NCC groups, the BPD and DD groups, as well as the DD and NCC groups in subsets of our data (e.g., for the comparison between the BPD and DD groups only responses from patients with BPD and DD were included). Results show that the descriptive between-group differences shown in Table 2 and discussed in the Results section reach statistical significance. Second, we repeated our main analyses using dynamic SEMs with single-item responses to indicate levels of feeling of worth and feeling worthless instead of composite scores for self-esteem. These analyses were motivated by our reliability analyses (see Method section), which indicated low within-person reliability of the composite score. Results from these analyses confirm our hypotheses tests (e.g., differences in trait self-esteem across diagnostic groups, as well as differences in the cross-lagged relation between arousal (t) and self-esteem (t + 1) across diagnostic groups). Interestingly, we found a significant negative cross-lagged relation between feelings of self-worth (t) and later arousal (t + 1), but no significant cross-lagged relation between feelings of worthless (t) and later arousal (t + 1). This might imply that momentary feelings of worth are more central for understanding the temporal association between self-esteem and arousal. Third, we repeated our main analysis using 30 and 60 min intervals between assessments. Results indicate no consistent pattern of cross-lagged relations between arousal and self-esteem at longer time intervals. Detailed results are provided in the open science framework depository.

## Discussion

The purpose of this study was to investigate the temporal relations between affective states and current self-esteem in patients with BPD. Assessing self-reports every 15 min, we found that higher ratings of affective arousal precede lower levels of momentary self-esteem in patients with BPD but not in patients with DD and NCC participants. In addition, we show that average daily (“trait”) levels of arousal are significantly higher, and average daily (“trait”) levels of self-esteem are significantly lower in patients with BPD than patients with DD, which, in turn, significantly differ from NCCs, as expected ([1]; also see [81]).

### Increases in arousal precede lower self-esteem in BPD

Results from this study resemble findings from existing experience sampling studies that found close associations between variation in momentary self-esteem and variation in affective states ([42, 70]; also see [67, 68]). We extend current evidence by investigating temporal dynamics using a dense sampling plan. Results indicate that higher scores in arousal are predictive of lower scores in self-esteem in BPD at short time intervals (ca. 15 min), whereas lower scores in self-esteem were not predictive of higher scores in arousal. This finding is consistent with the theoretical premise that current self-esteem (i.e., self-evaluations) in BPD varies as a function of (negative) affective states [23, 44]. Specifically, self-esteem instability might be partially explained by unfavorable self-evaluations following increases in negative affective states – a process that may be specific to BPD [70]. The fact that the temporal contingency between changes in affect and self-esteem seems to be rather short corresponds well with the dynamic instability in affect and self-esteem previously described in patients with BPD [66, 69]. At the same time, findings from this study do not align well with the idea that changes in current self-esteem (i.e., identity disturbances) in BPD lead to negative affective states such as helplessness – at least not in time intervals of 15, 30, and 60 min [38, 72]. Our results contradict one e-diary study showing that changes in self-esteem predict subsequent changes in levels of affective arousal among patients with BPD using hourly intervals [70]. We found no significant dynamic temporal relations between arousal states and current self-esteem in patients with DD or NCC participants. This null finding was somewhat unexpected in the DD group as current theories and evidence suggest temporal relations between unstable feelings of self-worth and depressive symptoms ([39, 56]; see [77] for a review). One reason for the different results is that temporal dynamics between self-esteem and negative affect in depression may occur at longer time intervals (e.g., days, weeks, or even months as opposed to minutes). For example, one typical study found that self-esteem instability predicted future depressive symptoms at a 6-months interval (Frank and De Raedt 2006 [10], also see Sowislo and Orth 2013 [76], [77]). During a depressive episode, mood and self-esteem are low for most individuals most of the time with little dynamic interaction at short time intervals. This is a noticeable difference to patients with BPD, who report frequent and drastic changes in affective states and current self-esteem that are closely related over time. Finally, our study adds to results from a previous e-diary study in which self-esteem instability was found to be higher in patients with BPD compared to patients with anxiety disorders, while levels of affective instability were comparable across diagnostic groups, leading the authors to conclude that self-esteem instability is a specific feature of BPD [42]. Identifying specific dynamics in psychopathology (e.g., involving self-esteem) is important as aspects of identity disturbance that may be unique to BPD could help to explain why patients with BPD are more likely to engage in dysfunctional behaviors to regulate negative affective states than, for example, patients with DD (e.g., [67, 68]).

### Autoregressive effects of affect and self-esteem

Results from our study suggest significant autocorrelations of moderate size for affect and self-esteem across diagnostic groups (BPD, DD, NCC) but no significant differences in the size of autocorrelations between groups. This finding means that all participants demonstrated a certain stability in their affect and self-esteem ratings from one time point to the next (15, 30, or 60 min later). The fact that the size of the autocorrelations does not significantly differ across diagnostic groups shows that on average arousal in patients with BPD is equally determined by prior arousal compared with patients with DD and NCC [16, 44]. The fact that the size of the autocorrelations does not significantly differ across diagnostic groups shows that on average arousal in patients with BPD is equally determined by prior arousal compared with patients with DD and NCC [16, 44]. This finding is in line with meta-analytic results indicating only a weak relation between the size of autocorrelations in negative affect and borderline symptoms [12].

### Limitations and future research

Although our study suggests specific dynamic temporal relations between arousal and self-esteem in patients with BPD, a few limitations should be mentioned. Because data for this study were collected as part of a larger data set, several of these limitations have already been discussed elsewhere [29, 30]. The first limitation is that experience samples were collected over the course of only 1 day and for patients with BPD in only one inpatient clinic. In the clinic, patients were removed from mundane activities and stressors and partly were undergoing treatment with dialectical behavioral therapy, whereas experience samples from most patients with DD and all NCC participants were collected at home. Treating self-criticism (or self-invalidation) and associated emotions such as shame and guilt is a main objective of dialectical behavioral therapy, and we cannot rule out that this influenced our results [45]. This limits the generalizability of our findings and complicates the comparisons of effects between diagnostic groups. We encourage future researchers to adopt a multi-centric approach, sample participants in similar settings (e.g., only patients in inpatient facilities or all participants during daily life), and assess constructs over longer periods (e.g., 2 weeks). The second limitation is that we have not provided contextual information to explain the changes in affective arousal, so it remains unclear which factors triggered changes in affective states and momentary self-esteem. Different triggers may result in different processes; for example, increased arousal due to unfavorable social comparisons, as well as negative or self-discrepant social feedback could be more likely to cause drops in self-esteem than other triggers are (see [34] for a review). In addition, different participant may vary in their reactivity to comparable (social) triggers (e.g., as a function of dispositional rejection sensitivity; [60]). Future studies should assess contextual information on the measurement occasion level and relevant explanatory variables on the person level (e.g., future researchers could include a measure of rejection sensitivity and use social stressor lists as implemented by Tolpin et al. 2014 [78]). This should help to identify instances in which affective states and momentary self-esteem change and allow for more detailed investigation of the circumstances. The third limitation is that a significant proportion of e-diary assessment data were missing because participants did not respond to prompts, and compliance was lowest in patients with BPD. One reason for this may be that participant burden was high given the dense sampling plan (every 15 min). Future research should implement procedures that enhance compliance (e.g., renumeration schemes, participant training, providing a method for suspending prompts in advance; [80]). Although we used a state-of-the-art analysis approach to account for missing responses and sensitivity analyses to ensure the robustness of our results, parameter estimates may be biased [4, 71]. Especially as our data set lacks variables that may explain why participants missed responses (e.g., therapy appointment, forgetfulness, etc.). Specifically, we cannot rule out that patients with BPD omitted prompts due to high levels of distress, which may have led to an underestimation of arousal levels. However, it is also possible that patients with BPD were more likely to respond to prompts in times of higher distress, which may have led to an overestimation of arousal levels. Future research should include variables that may explain missingness (e.g., conscientiousness ratings, activity data, retrospective distress ratings; [17]). The fourth limitation is that we used a single item measure to assess affect, and only two items to assess self-esteem. Although this approach helps to manage the burden placed on participants, especially during frequent assessments, there are drawbacks of this approach. There has been debate regarding the reliability of single item measures in experience sampling studies and most researchers recommend using at least three homogenous items per construct ([9, 17]; but also see [75]). The two-item measure we used to assess self-esteem had moderate within-level reliability. Future researchers should use more homogenous items to reliably capture within-person changes in self-esteem [9]. In addition, single item measures can only capture certain aspects of multifaceted psychological constructs and run the risk of insufficiently depicting key concepts [8]. For example, in this study we focused on a single outcome of identity disturbance (self-esteem), while neglecting other aspects of this complex construct such as self-knowledge and self-efficacy ([35]; also see [28, 53]). More so, the items we used to assess self-esteem differs from the items used in previous studies, which may result in different conceptualizations of the construct and complicate comparing results across studies (e.g., [42]. Future research would benefit from a validated scale designed to reliably capture within-person variability in self-esteem. Another limitation is that the BPD sample in this study is primarily female and reflects a treatment seeking population. In addition, we did not include a question on self-identified ethnicity, limiting evaluations of the diversity of our sample. Finally, future research should consider moving away from categorical diagnostic categories and test hypotheses regarding the interplay of affect and self-esteem in more homogenous groups (e.g., individuals with instable affects and/or self-esteem).

### Study implications for clinical practice

Despite these limitations, our results have useful implications for clinical interventions. For example, as we mentioned in a previous study [30], patients with BPD who report high levels of affective arousal should be trained to better understand their emotions and provided with functional strategies to regulate their arousal ([2], Linehan et al. 2014 [45]). Although further research (e.g., within-person randomized intervention studies) is needed to determine whether reducing levels of affective arousal helps to prevent drops in current self-esteem in patients with BPD, such an effect is at least plausible (also see [66, 69, 70]). Assisting patients with BPD in affective regulation is particularly important as recent research shows that in the presence of low levels of trait self-esteem, high levels of affective arousal precede dysfunctional behaviors such as nonsuicidal self-injury [67, 68]. Therapists who work with patients with BPD may also use interventions designed to bolster feelings of self-worth and teach emotion regulation strategies that promote self-kindness such as mindfulness, self-encouragement, and self-compassion [45, 54]; also see [19, 64]). Habitual positive self-evaluations should make it easier to become aware of and accept current affective states, interrupt downward spirals of rumination processes, and increases in negative affect and implement adaptive affect regulation strategies such as cognitive reappraisal [46, 73, 86].

## Conclusion

In sum, results from this study show that higher scores in affective arousal states precede lower scores in current self-esteem in patients with BPD (but not in clinical and non-clinical control participants), further emphasizing to the importance of affective (dys)regulation in BPD psychopathology [44]. Importantly, the reverse effect did not reach statistical significance, indicating that lower scores in current self-esteem do not precede higher affective arousal states [38].

### Supplementary Information

**Additional file 1: Table S1.** Main and Comorbid Mental Disorders of Patients in the BPD (*n* = 42) and DD (*n* = 40) Groups.

**Additional file 2.** Patient information about the study.

**Additional file 3.** Missing Data Patterns.

## Data Availability

The anonymized data set, statistical code, and additional results are available at: https://osf.io/8xwtc/.

## Footnotes

1 All removals in the BPD and NCC groups were female. In the DD group two males and six females were removed. The age range for removals was 21–48 years in the BPD group, 20–49 in the DD group, and 19–39 in the NCC group. Six out of seven (85.71%) removals in the BPD group and five out of eight (62.50%) removals in the DD group received psychotropic medication. Three out of seven (42.86%) removals in the BPD group had a comorbid PTSD diagnosis. The mean SCL-90R Global Severity Index scores for removals were 1.80 (*SD* = 1.07) in the BPD group, 1.60 (*SD* = 0.74) in the DD group, and 1.34 (*SD* = 0.43) in the NCC group.

2 Coefficient omega uses a confirmatory single-factor model to decompose the true (shared) and error (unshared) variances (see [49] for formulas). It denotes the proportion of the variance of the composite score due to the true score. The advantage of the coefficient omega over the more commonly reported coefficient alpha is that factor loadings in the underlying measurement model are not assumed to be equal across items but only that all items load on one common factor, which is a more tenable assumption in the context of our study. Hierarchical omega is reported because it is more general than coefficient omega, also accounting for variance due to correlations between error terms (see [49], Kelly and Pornprasertmanit 2016 for formulas and a discussion [37]).

3 Experience sampling leads to observed scores that can be decomposed in true (shared) and error (unshared) variances at both the within- and the between-levels. Multilevel composite reliability uses a multilevel factor model to decompose true (shared) and error (unshared) variances at both levels (see Lai 2021 for formulas [43]). The overall reliability coefficient denotes the proportion of the variance of the composite score due to the true score on the within- and between-levels (overall), whereas the within-level coefficient only considers the within-level and the between-level coefficient only considers the between-level (also see [25]).

4 Average differences between groups are equidistant because we used one grouping variable (1 = BPD, 2 = DD, 3 = NCC) as a predictor. For example, the estimated difference in arousal between the BPD and the NCC groups is 2*(-1.98) = -3.96. Multigroup models for dynamic structural equation models would have allowed us to test exact group differences for significance within a single model. At the time we ran our analysis, however, such analyses were not available in Mplus. We computed additional models including dummy-coded variables to investigate group differences (see our Sensitivity and additional analyses section).
