# Supplementary material for: Increases in negative affective arousal precede lower self-esteem in patients with borderline personality disorder but not in patients with depressive disorders: an experience sampling approach
Source: Borderline Personal Disord Emot Dysregul. 2023 Oct 3;10:29. doi: 10.1186/s40479-023-00229-w (PMC10546701; doi:10.1186/s40479-023-00229-w)
Supplement: Supplementary file 1 — Additional file 1: Table S1. Main and Comorbid Mental Disorders of Patients in the BPD (n = 42) and DD (n = 40) Groups. [file 40479_2023_229_MOESM1_ESM.pdf]

Table S1

Main and Comorbid Mental Disorders of Patients in the BPD (n = 42) and DD (n = 40) Groups

	BPD		DD	
	n	%	n	%
**Main diagnosis (ICD-10; DSM-IV)**				
Borderline personality disorder (F60.31; 301.83)	42	100%	0	0%
Mild depressive episode (F32.0; 296.21)	0	0%	1	2.50%
Moderate depressive episode (F32.1; 296.22)	0	0%	12	30.00%
Recurrent depressive disorder, current episode mild (F33.0; 296.31)	0	0%	1	2.50%
Recurrent depressive disorder, current episode moderate (F33.1; 296.32)	0	0%	15	37.50%
Recurrent depressive disorder, current episode severe without psychotic symptoms (F33.2; 296.33)	0	0%	4	10.00%
Dysthymia (F34.1; 300.4)	0	0%	7	17.50%
**Comorbid diagnoses (ICD-10; DSM-IV)**				
Harmful use of alcohol (F10.10; 305.00)	1	2.38%	0	0%
Harmful use of cannabinoids (F12.10; 305.20)	1	2.38%	0	0%
Dependence on sedatives or hypnotics (F13.20; 304.10)	1	2.38%	0	0%
Recurrent depressive disorder, current episode moderate (F33.1; 296.32)	1	2.38%	0	0%
Recurrent depressive disorder, currently in remission (F33.4; 296.36)	23	54.76%	0	0%
Agoraphobia (F40.0; 300.22)	0	0%	1	2.50%
Agoraphobia with panic disorder (F40.01; 300.21)	0	0%	1	2.50%
Social anxiety disorder (F40.1; 300.23)	3	7.14%	1	2.50%
Panic disorder (F41.0; 300.01)	0	0%	2	5.00%
Obsessive-compulsive disorder, predominantly obsessive thoughts (F42.0; 300.3)	1	2.38%	0	0%
Obsessive-compulsive disorder, predominantly compulsive acts (F42.1; 300.3)	1	2.38%	1	2.50%
Post-traumatic stress disorder (F43.1; 309.81)	14	33.33%	4	10.00%
Dissociative convulsions (F44.5; 300.11)	0	0%	1	2.50%
Somatization disorder (F45.0; 300.81)	0	0%	1	2.50%
Atypical anorexia nervosa (F50.1; 307.10)	1	2.38%	0	0%
Bulimia nervosa (F50.2; 307.51)	3	7.14%	1	2.50%
Other eating disorders (F50.8; 307.51)	2	4.76%	0	0%
Eating disorder, unspecified (F50.9; 307.50)	5	11.9%	2	5.00%
Schizoid personality disorder (F60.1; 301.20)	1	2.38%	0	0%
Anxious personality disorder (F60.6; 301.82)	2	4.76%	2	5.00%
Dependent personality disorder (F60.7; 301.6)	1	2.37%	0	0%
Mixed and other personality disorders (F61.0; 301.9)	1	2.38%	0	0%
ADHD (F90.0; 314.0)	3	7.14%	1	2.50%

Note. Assessed using structured clinical interviews based on DSM-IV diagnostic criteria. BPD = borderline personality disorder; DD = depressive disorders.
