# Supplementary material for: Increases in negative affective arousal precede lower self-esteem in patients with borderline personality disorder but not in patients with depressive disorders: an experience sampling approach
Source: Borderline Personal Disord Emot Dysregul. 2023 Oct 3;10:29. doi: 10.1186/s40479-023-00229-w (PMC10546701; doi:10.1186/s40479-023-00229-w)
Supplement: Supplementary file 2 — Additional file 2. Patient information about the study. [file 40479_2023_229_MOESM2_ESM.pdf]

Patient information about the study

Version 6.1.2016

"Assessment of tension using a smartphone app in patients with borderline personality disorder (BPD) and clinical and non-clinical controls"

Dear prospective participant,

in this patient information leaflet, we would like to explain the study project to you.

Introduction

We hereby offer you the participation in a scientific study. In this study, the Charité will evaluate pseudonymized data that will be collected by the company Bluebird Technology using a smartphone app. With your consent to this study, we may receive pseudonymized data from the company Bluebird Technology and will use it for scientific purposes. The company Bluebird Technology collects the data for itself and not on behalf of Charité. Bluebird Technology is not part of Charité. The company Bluebird Technology will provide you with a smartphone. Bluebird Technology will collect pseudonymized data via the app, which means that the company Bluebird technology cannot trace the data back to you personally. The data is made available to the Charité via a secure connection. The provisions formulated in the smartphone app between you and Bluebird apply to the processing of the data up to the point of transmission. You will be assigned a personal code, which you will receive from Bluebird Technology. This code allows to link the data in the app with data collected at Charité. All further processing takes place in pseudonymized form. The data collection process consists of three parts. In the first part, during your waking hours over the course of one day (24 hours), you will be asked regularly via the smartphone app regarding certain symptoms, thoughts, emotions and intentions. The app data are collected and pseudonymized by the company Bluebird Technology. At this point you will receive a personal code. The data are then transmitted to Chartité in pseudonymized form through a secure connection. Your personal code is used to link the data collected by Bluebird Technoligy and the socio-demographic and psychometric data (i.e. age, gender, diagnoses, severity of symptoms, medication) collected at Charité. Data are only transmitted to Bluebird Technology in anonymized form. The exact study procedure is described below.

Purpose of the study

The aim of this study is to understand whether certain symptoms are related to perceived stress and whether stressors or symptom can be changed using emotion regulation strategies.

Procedure and duration of participation

If you do not have the technical means, a device will be made available to you. The data are collected using a smartphone app developed by Bluebird Technology and then made available to the Charité. You will be thoroughly instructed on how to recognize and report the symptoms, thoughts, emotions and intentions that are to be examined. Subsequently, you will be asked to make an assessment of the previously defined items every 10-20 minutes during the waking hours of a day. A short signal (ringing or vibration) will alert you to the entry. If an entry is not possible at this time, you can reject it. In addition to

assessments described above, physiological data are collected using sensors in the smartphones during over the survey period (24 hours).

Possible risks

All screening interventions are non-invasive and well tolerable. The performance of all tasks in this study is risk-free when compliance and exclusion criteria are met. Despite all measures taken by the company Bluebird Technology and by the Charité, it must be pointed out that safety gaps may exist. For example, since data transmission runs through WIFI or commercial telephone networks, the security is limited to the general safety of data transfer in these networks and we cannot role out that information are intercepted by third parties.

Possible benefit for the public and study participants

The planned study is a human trial (experiment) that is not intended to cure diseases. Assessments with extremely low health risk are carried out for scientific purposes. Only certified devices will be used, whose guidelines for use will be strictly followed. You yourself as a study participant will not have any direct benefit from this study.

Data protection

The data to be collected using the smartphone app will be stored in pseudonymized form by Bluebird Technology with your consent. If you hereby consent to the transfer of the data to and processing by the Charité, Bluebird Technology will make your data available to the Charité in pseudonymized form via a secure connection. At Charité, this pseudonymized data are linked to personal data collected at Charité, using the personal code assigned by Bluebird Technology. The processing of the data is then again pseudonymized. Only authorized persons (Prof. Dr. Röpke and two members of the working group) have access to the data via two-factor authentication. Data will be stored on the Charité server according to standardized procedures, in standard filing cabinets and with authorization groups (people who work in the project). The data storage is kept separately from the data collected at Charité in a secure filing cabinet. Only the study director, Prof. Dr. Röpke, has access to this cabinet. Third parties, including Bluebird Technologies, have no access. Electronic processing takes place with the pseudonymized data. By signing this consent form, you agree that the study doctor and his staff may collect and process your personal data for the purpose of the above-mentioned study. Personal data are, for example, your date of birth, gender, ethnicity, data about your physical and mental health, as well as other personal data collected during this study. The study doctor will use your personal data for the purposes of administering and conducting the study, as well as for the purposes of research and statistical analysis. The data will be processed and stored in encrypted form. For this purpose, the data will be provided with a code number (pseudonymization of the data).

You have the right to access all your personal data held by the study doctor. You also have the right to have any inaccuracies in your personal data corrected. If you wish to make a request, please contact the study director. You will find the address and telephone number of the study doctor at the end of the form. Please note that the results of the study will be published in the medical literature. Your identity will remain anonymous. You can object at any time to the further processing of your data collected in the context of the above-mentioned study and demand that it be deleted or destroyed. Data will be archived for 10

years (see §7 of the Charité statutes on safeguarding scientific practice). Please keep in mind that once the de-pseudonymization list has been deleted, it is no longer possible to delete single entries in the data set.

Insurance coverage

We would like to inform you that no separate insurance has been taken out for you. The doctors and scientists involved in the study are only insured against liability claims that could result from your culpable behavior through the company liability insurance.

Obligation to notify

You are obliged to follow the instructions of the study director for the duration of your participation in the study and to inform him immediately of any changes in your health.

Voluntary participation and withdrawal

Your participation in this study is voluntary. If you choose not to participate in this study, you will not suffer any disadvantages. The same applies if you withdraw your participation during the study. You can terminate your participation at any time during the study. You do not have to justify a possible revocation of your consent or withdrawal from the study.

Expense allowance

You will face no personal costs as a result of this study. Travel costs can be reimbursed. No further financial compensation is provided.

Contact person and right to ask questions

We would like to point out that you naturally have the right to contact the director of studies at any time regarding all study matters. Questions should be addressed to

Informed consent for the study

6.1.2016

Declaration of consent to participate in the study “Assesment of tension using a smartphone app in patients with borderline personality disorder (BPD) and clinical and non-clinical controls”

I hereby declare,

Name:

First name:

Address:

Date of birth:

that I have been informed orally and in writing by Mr/Mrs______________________ about the nature, significance, implications and risks of the scientific investigation within the scope of the above-mentioned study and have had sufficient opportunity to clarify my questions in this regard in a discussion with the study doctor.

I have understood the participant information sheet dated 06.01.2016. The sheet has been presented to me and I have received a copy of it together with a copy of this declaration of consent. I am aware that I can withdraw my consent at any time without giving reasons for doing so. No adverse consequences will follow. I understand that I can object to further processing of my data at any time and demand deletion of my personal data.

I am willing to participate in the scientific investigation within the framework of the above-mentioned study.

____

Date, place, signature

I agree that in the context of this study, personal data/information concerning me may be recorded and processed by the study doctor in a pseudonymized form on electronic data carriers. I also agree that the study results may be published in an anonymous form that does not allow any conclusions to be drawn about the person.

____

(Signature of the test participant)

I hereby declare that I have informed the above-named participant at _______________ verbally and in writing about the nature, significance, scope and risks of the above-named study and have handed over the copy of the information and this consent form to him/her.

____

(Signature of the informing investigator)

Patienteninformation zur Studie

Version 6.1.2016

Anspannungserfassung mittels Smartphone- App bei Patienten bei Borderline- Persönlichkeitsstörung (BPS) und klinischen und nicht-klinischen Kontrollen

Sehr geehrte Studieninteressentin, sehr geehrter Studieninteressent,

in der Ihnen vorliegenden Patienteninformation möchten wir Ihnen das Studienvorhaben erläutern.

Einleitung

Hiermit bieten wir Ihnen die Teilnahme an einer wissenschaftlichen Studie an. In dieser Studie werden an der Charité pseudonymisierte Daten, die Sie vorher durch die Firma Bluebird Technology mittels Smartphone App haben erheben lassen, ausgewertet. Mit Ihrer Zustimmung zu dieser Studie dürfen wir diese pseudonymisierten Daten von der Firma Bluebird Technology (Dritte Stelle) erhalten und verwenden. Die Firma Bluebird Technology erhebt die Daten für sich selbst und nicht im Auftrag der Charité, Bluebird Technology ist auch nicht Teil der Charité. Die Firma Bluebird Technology wird Ihnen eine Smartphone- App zur Verfügung stellen. Über das Smartphone-App wird Bluebird Technology pseudonymisierte Daten erheben, d.h. für die Firma Bluebird Technology sind die Daten nicht auf Ihre Person rückführbar. Über eine gesicherte Verbindung werden die Daten der Charité zur Verfügung gestellt. Für die Verarbeitung der Daten bis zum Zeitpunkt der Übermittlung gelten die in der Smartphone-App formulierten Bestimmungen zwischen Nutzer und Bluebird. Nur innerhalb der Charité ist über einen persönlichen Code, den Sie von der Firma Bluebird Technology erhalten werden, die Zuordnung zu Ihrer Person möglich und die Daten können in der Charité erhobenen personenbezogenen Daten zugeordnet werden. Die weitere Verarbeitung erfolgt wieder in pseudonymisierter Form. Bluebird Technology erhebt die Daten für die Studie. Es werden in Ihrer Wachzeit über einen Tag (24 Stunden) in regelmäßigen Abständen Abfragen über eine Smartphone-App zu bestimmten Symptomen, Gedanken, Gefühlen und Handlungsintensionen von Ihnen gemacht. Die App-Daten werden von der Firma Bluebird Technology erhoben und Ihnen wird ein Code mitgeteilt. Anschließend werden die Daten in pseudonymisierter Form durch eine gesicherte Verbindung an die Charité übermittelt. Über den Code erfolgt an der Charité eine Zuordnung zu Ihren soziodemografischen und psychometrischen Daten (d.h. Alter, Geschlecht, Diagnosen, Schweregrad der Symptome, Medikation). Daten werden an die Firma Bluebird Technology nur in anonymisierter Formübermittelt. Im Folgenden wird Ihnen der genaue Studienablauf geschildert.

Zweck der Studie

Ziel dieser Studie ist es zu verstehen, ob bestimmte Symptome mit der gefühlten Belastung zusammenhängen.

Ablauf und Dauer der Teilnahme

Auf Ihr Smartphone haben Sie durch die Firma Bluebird Technology eine App installieren lassen, mit der die Untersuchung durchgeführt werden kann. Sollten Sie kein Smartphone

besitzen oder Ihr Gerät nicht über die technischen Voraussetzungen verfügen kann Ihnen ein Gerät zur Verfügung gestellt werden. Die Daten werden mittels Smartphone-App von der Firma Bluebird Technology erhoben und anschließend der Charité zur Auswertung zur Verfügung gestellt. Sie werden genau instruiert, wie Sie bei sich die zu untersuchenden Symptome, Gedanken, Gefühle und Handlungsintentionen erkennen und einschätzen können. Im Anschluss werden Sie über die Wachzeit an einem Tag alle 10-20 Minuten gebeten die Einschätzungen hinsichtlich der vorher festgelegten Items zu treffen. Durch ein kurzes Signal (Klingeln oder Vibration) werden Sie auf die Eingabe hingewiesen. Sollte eine Eingabe zu diesem Zeitpunkt nicht möglich sein kann diese von Ihnen abgelehnt werden. Neben der von Ihnen getätigten Eingabe werden auch die oben beschriebenen physiologischen Daten mittels Sensoren über den Erhebungszeitraum (24 Stunden) gesammelt.

Mögliche Risiken

Sämtliche Untersuchungsmethoden sind nicht-invasiv und durch eine gute Verträglichkeit gekennzeichnet. Die Durchführung aller in der Studie verwendeten Aufgaben sind bei Einhaltung der Ein- und Ausschlusskriterien ohne Risiken. Trotz aller, außerhalb der Charité von der Firma Bluebird Technology, innerhalb der Charité von der Charité selbst getroffenen Maßnahmen zum Schutz der Daten muss darauf hingewiesen werden, dass Sicherheitslücken bestehen können. Z.B.: Da die Datenübertragung durch das WLAN- Netz bzw. kommerzielle Telefonnetze läuft gilt wie bei diesen Kommunikationsnetzwerken generell, dass die Sicherheit eingeschränkt ist und die Inhalte von Dritten abgefangen („mitgehört“) werden könnten.

Möglicher Nutzen für die Allgemeinheit bzw. den Studienteilnehmer

Bei der geplanten Studie handelt es sich um einen Humanversuch (Experiment) mit Studienteilnehmern ohne Heilversuch. Es werden Messungen mit äußerst geringem gesundheitlichem Risiko für wissenschaftliche Zwecke durchgeführt. Dabei werden nur zertifizierte Geräte verwendet, deren Richtlinien zur Anwendung genauestens Folge geleistet wird. Sie selber als Studienteilnehmer werden keinen unmittelbaren Nutzen an dieser Studie haben.

Datenschutz

Die per Smartphone-App zu erhebenden Daten werden mit Ihrer Zustimmung von der Firma Blue Bird Technologies pseudonym erhoben und gespeichert. Stimmen Sie hiermit der Daten an und der Verarbeitung durch die Charité zu, stellt Blue Bird die Daten in pseudonymisierter Form über eine gesicherte Verbindung der Charité zur Verfügung. In der Charité werden diese pseudonymisierten Daten über den von der Firma Blue Bird Technology vergebenen Code Ihrer Person zugeordnet und mit den an der Charité erhobenen personenbezogenen Daten verbunden. Die Verarbeitung der Daten erfolgt dann wieder pseudonymisiert. Es haben nur berechtigte Personen (Prof. Dr. Röpke und zwei Mitarbeiter der Arbeitsgruppe) per Zwei-Faktor-Authentifizierung Zugriff. Die Datenspeicherung findet auf dem Charité-Server nach standardisiertem Verfahren statt, in Standardablagen und mit Berechtigungsgruppen (Personen, die im Projekt arbeiten). Die Schlüssel zur Entpseudonymisierung werden von der Charité separat von den Daten in einem gesicherten Aktenschrank verwahrt. Lediglich der Studienleiter, Prod. Dr. Röpke, hat Zugriff auf diesen gesicherten Aktenschrank. Dritte, d.h. auch Bluebird Technologies, haben

hierauf keinen Zugriff. Eine elektronische Verarbeitung findet nur mit pseudonymisierten Daten statt. Durch Ihre Unterschrift auf der Einwilligungserklärung erklären Sie sich damit einverstanden, dass der Studienarzt und seine Mitarbeiter*innen Ihre personenbezogenen Daten zum Zweck der oben genannten Studie erheben und verarbeiten dürfen. Personenbezogene Daten sind z.B. Ihr Geburtsdatum, Ihr Geschlecht, Ihre ethnische Zugehörigkeit, Daten zu Ihrer psychischen und physischen Gesundheit oder andere persönliche Daten die während Ihrer Teilnahme an dieser Studie erhoben wurden. Der Studienarzt wird Ihre personenbezogenen Daten für Zwecke der Verwaltung und Durchführung der Studie verwenden, ebenso für Zwecke der Forschung und statistischen Auswertung. Die Daten werden dabei in verschlüsselter Form verarbeitet und gespeichert. Hierzu werden die Daten mit einer Codenummer versehen (Pseudonymisierung der Daten). Sie haben das Recht auf Auskunft über alle beim Studienarzt vorhandenen personenbezogenen Daten über Sie. Sie haben auch Anrecht auf Korrektur eventueller Ungenauigkeiten in Ihren personenbezogenen Daten. Wenn Sie eine Anfrage machen wollen, wenden Sie sich bitte an die Studienleiter. Die Adresse und Telefonnummer des Studienarztes finden Sie am Ende dieses Formblatts. Bitte beachten Sie, dass die Ergebnisse der Studie in der medizinischen Fachliteratur veröffentlicht werden können, wobei Ihre Identität jedoch anonym bleibt. Sie können jederzeit der Weiterverarbeitung Ihrer im Rahmen der o.g. Studie erhobenen Daten widersprechen und ihre Löschung bzw. Vernichtung verlangen. Ansonsten werden die Daten, wie gesetzlich vorgeschrieben, für 10 Jahre archiviert (§7 der Satzung der Charité zur Sicherung guter wissenschaftlicher Praxis). Bitte bedenken Sie, dass eine Löschung der kompletten Daten, nach erfolgtem Widerruf nicht mehr umsetzbar ist, sobald die Entpseudonymisierungsliste gelöscht wurde.

Versicherungsschutz

Wir möchten Sie darüber aufklären, dass keine seperate Versicherung für Sie abgeschlossen worden ist. Die an der Studie beteiligten Ärzte und Wissenschaftler sind lediglich durch die Betriebshaftpflichtversicherung gegen Haftungsansprüche, welche aus ihrem schuldhaften Verhalten resultieren könnten, versichert.

Mitteilungspflicht

Sie sind verpflichtet während der Dauer der Versuchsteilnahme den Anweisungen des Studienleiters zu folgen und diesen über Veränderungen Ihres gesundheitlichen Wohlbefindens unverzüglich zu informieren.

Freiwillige Teilnahme und Rücktritt

Ihre Teilnahme an dieser Studie ist freiwillig. Wenn Sie auf die Teilnahme an dieser Studie verzichten, haben Sie keine Nachteile zu erwarten. Das gleiche gilt, wenn Sie Ihre Einwilligung zu einem späteren Zeitpunkt widerrufen. Die Möglichkeit, Ihre Teilnahme zu beenden, haben Sie jederzeit während der Studie. Einen allfälligen Widerruf Ihrer Einwilligung bzw. den Rücktritt von der Studie müssen Sie nicht begründen.

Aufwandsentschädigung

Durch diese Studie entstehen für Sie keine Kosten. Fahrtkosten können Ihnen bei Vorlage des Nachweises erstattet werden. Eine weitere finanzielle Entschädigung ist nicht vorgesehen.

Ansprechpartner und Fragerecht

Wir möchten darauf hinweisen, dass Sie selbstverständlich jederzeit das Recht haben den Studienleiter zu sämtlichen Studienangelegenheiten zu kontaktieren.

Einwilligungserklärung zur Studie

6.1.2016

Einwilligungserklärung über die Teilnahme an der Studie „Anspannungserfassung mittels Smartphone-App bei Patienten mit Borderline-Persönlichkeitsstörung (BPS) und klinischen und nicht-klinischen Kontrollen“

Hiermit erkläre ich,

Name:

Vorname:

Anschrift:

Geburtsdatum:

dass ich durch Herrn/Frau _______________________ mündlich und schriftlich über das Wesen, die Bedeutung, Tragweite und Risiken der wissenschaftlichen Untersuchung im Rahmen der o.g. Studie informiert wurde und ausreichend Gelegenheit hatte meine Fragen hierzu in einem Gespräch mit dem Studienarzt zu klären.

Ich habe insbesondere die mir vorgelegte Teilnehmerinformation vom 06.01.2016 verstanden und eine Ausfertigung derselben und dieser Einwilligungserklärung erhalten. Mir ist bekannt, dass ich meine Einwilligung jederzeit ohne Angabe von Gründen und ohne nachteilige Folgen für mich zurückziehen kann und eine Weiterverarbeitung meiner Daten jederzeit widersprechen und ihre Löschung bzw. Vernichtung verlangen kann.

Ich bin bereit, an der wissenschaftlichen Untersuchung im Rahmen der o.g. Studie teilzunehmen.

____

Datum, Ort, Unterschrift

Einwilligungserklärung zur Datenerhebung und Datenverarbeitung

Ich erkläre mich damit einverstanden, dass im Rahmen dieser Studie mich betreffende personenbezogene Daten/ Angaben durch den Studienarzt erhoben pseudonymisiert auf elektronischen Datenträgern aufgezeichnet und verarbeitet werden dürfen. Ich bin auch damit einverstanden, dass die Studienergebnisse in anonymer Form, die keinen Rückschluss auf meine Person zulassen, veröffentlicht werden.

____

Unterschrift des Studienteilnehmers bzw. der Studienteilnehmerin

Hiermit erkläre ich den o.g. Teilnehmer am ___________________ über Wesen, Bedeutung, Tragweite und Risiken der o.g. Studie mündlich und schriftlich aufgeklärt und ihm eine Ausfertigung der Information sowie dieser Einwilligungserklärung übergeben zu haben.

____

(Unterschrift des aufklärenden Prüfarztes)
