# Supplementary material for: Increases in negative affective arousal precede lower self-esteem in patients with borderline personality disorder but not in patients with depressive disorders: an experience sampling approach
Source: Borderline Personal Disord Emot Dysregul. 2023 Oct 3;10:29. doi: 10.1186/s40479-023-00229-w (PMC10546701; doi:10.1186/s40479-023-00229-w)
Supplement: Supplementary file 3 — Additional file 3. Missing Data Patterns. [file 40479_2023_229_MOESM3_ESM.pdf]

Missing Data Patterns

We carefully inspected at which times data were missing across diagnostic groups (BPD, DD, NCC). The figures below illustrate the number of missing values at different times of the day. For example, in the BPD group only 4 out of 42 participants responded to the first prompt at 9 AM. The data displayed in the figures below suggests that across groups most responses were missed in the morning.

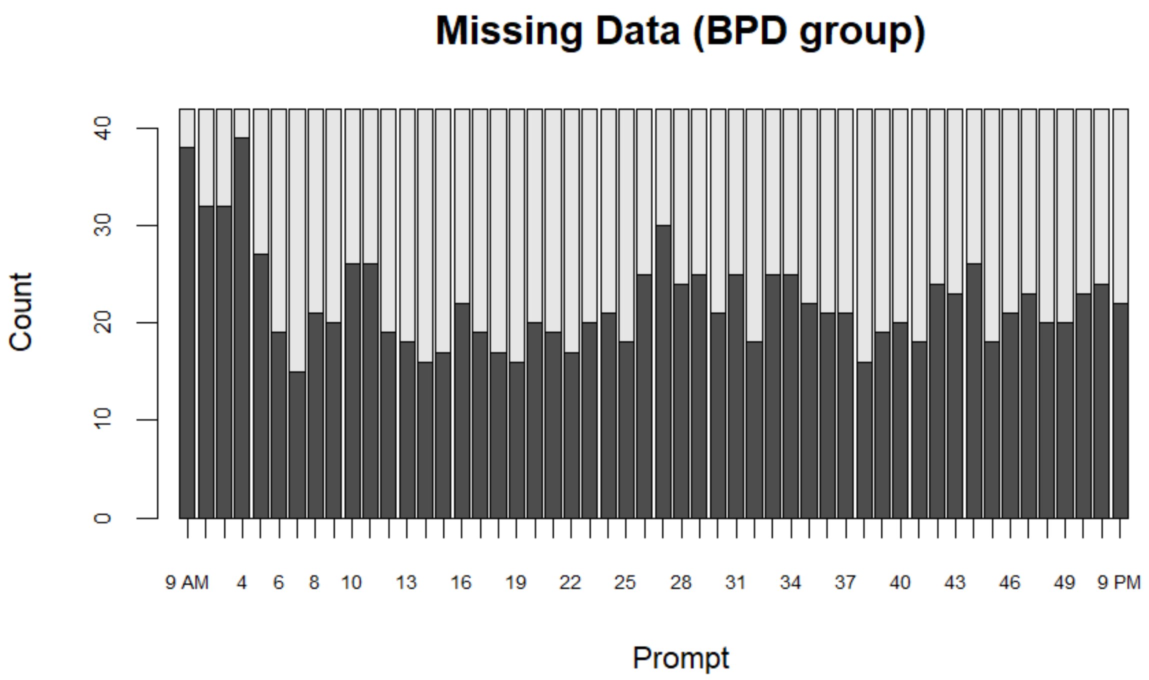

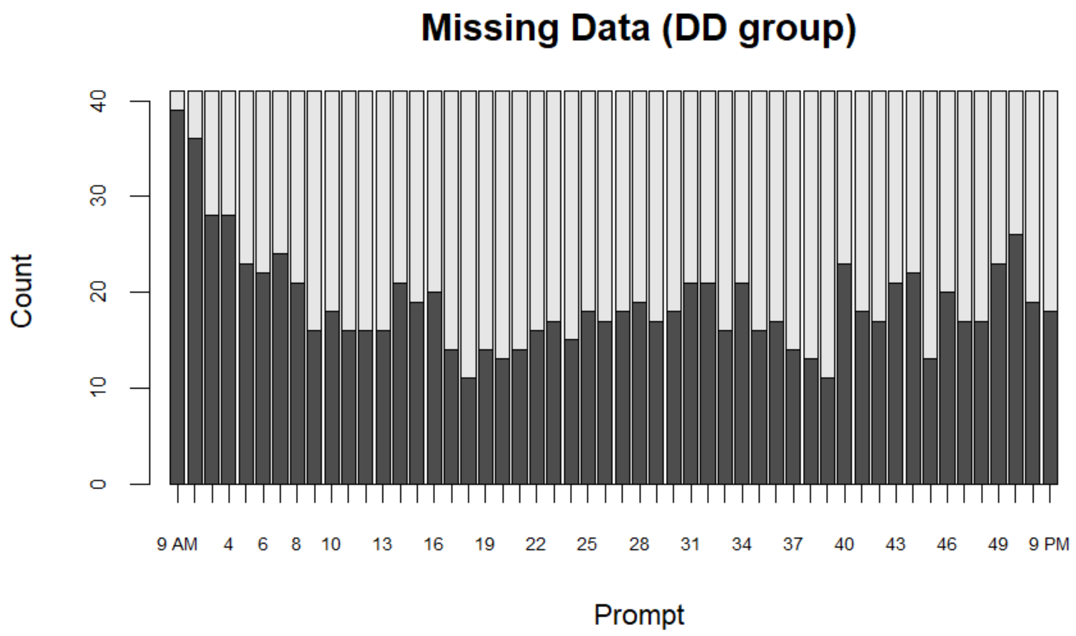

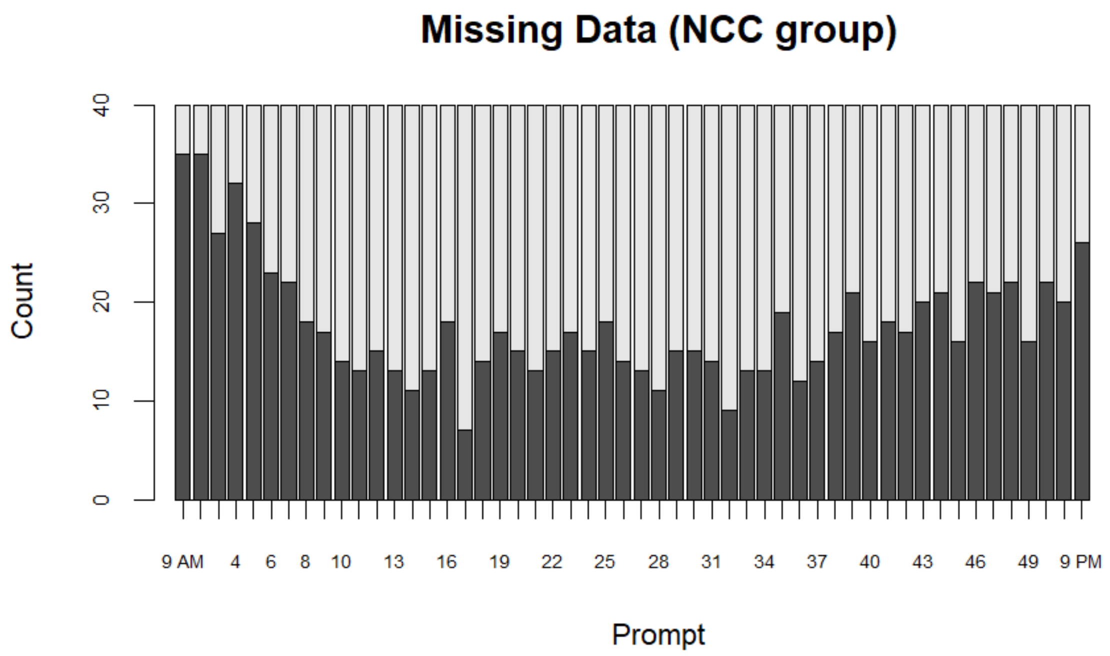
Missing Data (NCC group)
Count
0
10
20
30
40
9 AM
4
6
8
10
13
16
19
22
25
28
31
34
37
40
43
46
49
9 PM
Prompt
